# Medial Cuneiform Opening Wedge Osteotomy for Correction of Flexible Flatfoot Deformity: Trabecular Titanium vs. Bone Allograft Wedges

**DOI:** 10.1155/2019/1472471

**Published:** 2019-03-31

**Authors:** Giovanni Romeo, Alberto Bianchi, Vincenzo Cerbone, Matteo Maria Parrini, Francesco Malerba, Nicolò Martinelli

**Affiliations:** ^1^Department of Ankle and Foot Surgery, ICCS Istituto Clinico Città Studi, 20131 Milan, Italy; ^2^Department of Ankle and Foot Surgery, IRCCS Istituto Ortopedico Galeazzi, 20145 Milan, Italy; ^3^Orthopaedic Institute, ASST Pini/CTO, University of Milan, 20122 Milan, Italy

## Abstract

Adult flatfoot is a common pathology characterized by multiplanar deformity involving hindfoot, midfoot, and forefoot. Various surgical techniques have been described for the treatment but may not adequately correct the fixed forefoot varus component. Residual forefoot supination can be addressed by a plantar flexing opening wedge osteotomy of the medial cuneiform, also known as a Cotton osteotomy. Thus, the aims of this study were to compare clinical, radiological, and functional outcome after Cotton osteotomy, in patients treated with bone allograft or metallic implant. Consequently, 36 patients treated with opening wedge osteotomy of the medial cuneiform for forefoot varus were studied retrospectively. Patients were divided into two groups: the bone allograft group (HBG) (n=18) and the metallic implant group with BIOFOAM® Cotton Wedges (TTW) (n=18). Radiographic assessment and clinical scores including American Orthopaedic Foot and Ankle Society score, Foot Function Index, and visual analogue scale for pain were collected before operation and the last follow-up. The difference between baseline and follow-up for both groups was statistically significant for all the clinical scores and radiographic angles (p < 0.05). Most participants (92%) were very satisfied after surgery. Our results showed that Cotton osteotomy with a metallic implant provided both good clinical and radiographic outcomes comparable with bone allograft.

## 1. Introduction

Flatfoot is a common condition in children, adolescents, and some adults. During first years of life it is usually bilateral and asymptomatic [1]. In adults, the typical loss of the longitudinal arch is associated with deformity which may involve all three planes and different joints: hindfoot, midfoot, and forefoot with subjective and functional abnormalities [2]. Deformities become symptomatic when functional overload causes a deficiency in the muscle-tendon complex resulting in joint subluxation with sense of instability, fatigue and pain [3]. When conservative treatment is ineffective, surgical correction provides good clinical outcomes [4, 5]. Different surgical techniques including calcaneal osteotomies, subtalar arthrodesis, and tendon transfer have been proposed for the correction of the deformity, but often forefoot varus component should be managed with an adjunctive procedure. Residual forefoot varus can be addressed by a plantar flexing opening wedge osteotomy of the medial cuneiform, also known as a Cotton osteotomy [6]. This procedure is performed by placing an autologous (autograft) or homologous (allograft) wedge-shaped bone graft inside the medial cuneiform [7]. During recent years, trabecular titanium wedges have been proposed in substitution of the bone grafts to fill the gap during medial opening wedge osteotomy. The aim of the present study is to compare clinical and radiographic outcomes of patients who underwent Cotton osteotomy with allograft or trabecular titanium wedges.

## 2. Material and Methods

From November 2013 to January 2017, 36 of consecutive Cotton osteotomies and medializing calcaneal osteotomies (MCO) were performed by the senior surgeon (F.M.) in 36 patients (13 males [36.1%] and 23 females [63.9%]) with a mean age of 35.7 (range 14-64) years at the time of the procedure [Table 1]. The consecutive patients were allocated nonrandomly into two groups according to the date of surgery between 2013 and 2017: the first group received homologous bone graft (HBG) (n=18) from 2013 to 2015, and the second group received metallic implant (TTW: titanium trabecular wedge) with BIOFOAM® (Wright Medical Technology, Inc, Arlington, TN) Cotton Wedges (n=18) from 2015 to 2017. The study was carried out in accordance with the World Medical Association Declaration of Helsinki and all the patients provided written informed consent for inclusion. Patients were considered suitable candidates for this flatfoot correction procedure if they had the following conditions:

- Intractable hindfoot pain refractory to conservative treatment with symptoms lasting more than 12 months
- Collapse of longitudinal arch, hindfoot valgus (>5°), and forefoot varus (>15°) confirmed by clinical and radiological examination
- Flexible flatfoot deformity

Patients were excluded in case of tabagism, diabetes, and rheumatoid arthritis.

### 2.1. Surgery Technique

All the surgical procedures were performed by a single surgeon (F.M.) using two surgical position: lateral decubitus and supine position [8]. Antibiotic prophylaxis (a second-generation cephalosporin) was given preoperatively as a single dose. A midthigh pneumatic tourniquet was used on the ipsilateral extremity, and the calcaneus is exposed through an oblique incision in line with the osteotomy and is made parallel to the posterior facet of the subtalar joint and 2 cm posterior to it. The peroneal tendons and the sural nerve were reflected proximally, and the periosteum tissue is removed to expose the site of osteotomy. MCO was performed with an oscillating saw inclined posteriorly approximately 45 degrees to the plantar surface of the hindfoot. Once the osteotomy was completed, the posterior fragment is shifted 1 cm medially [9]. The osteotomy was secured with 2 parallels K-Wires or with a 7.3 mm cannulated screw.

**Figure 1:**
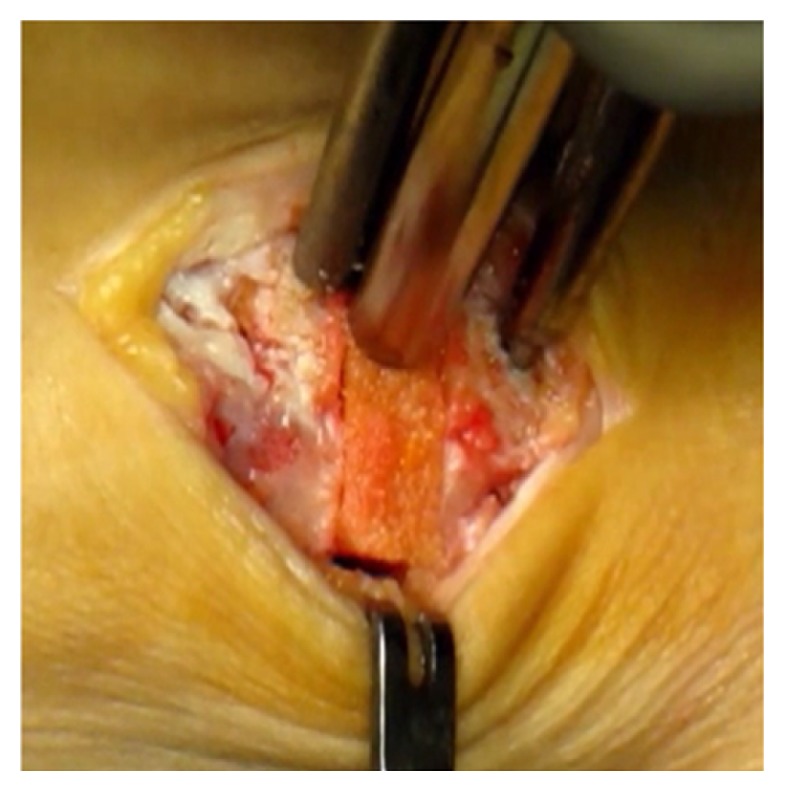
Cotton osteotomy with homologous bone graft taken from cadaver (Fresh frozen allograft).

**Figure 2:**
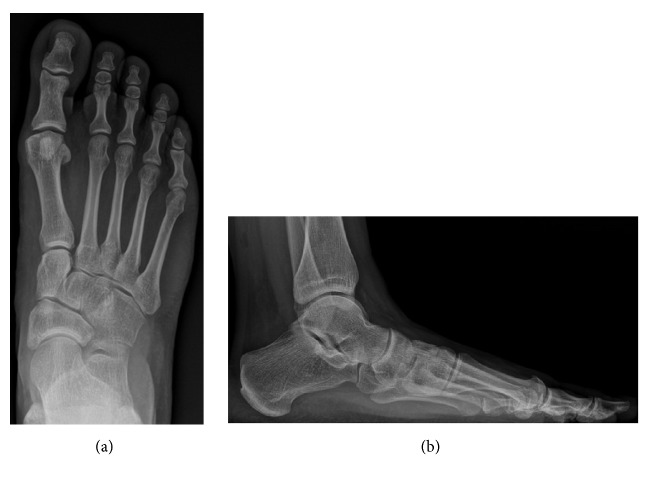
Preoperative anterior-posterior (a) and lateral (b) weight-bearing radiographs.

**Figure 3:**
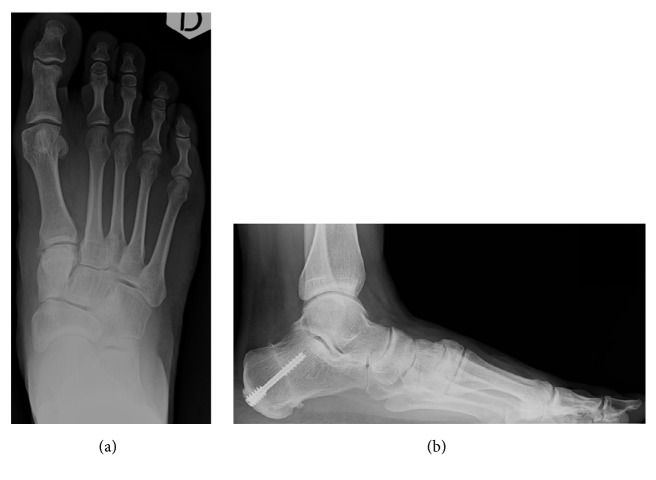
Postoperative (bone allograft) anterior-posterior (a) and lateral (b) weight-bearing radiographs.

**Figure 4:**
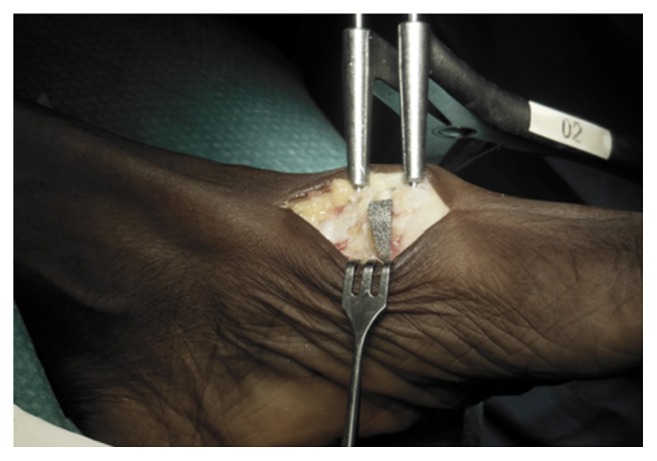
Cotton Osteotomy with trabecular Titanium Wedge (BIOFOAM-Cancellous Titanium™ Wedge-Wright®).

**Figure 5:**
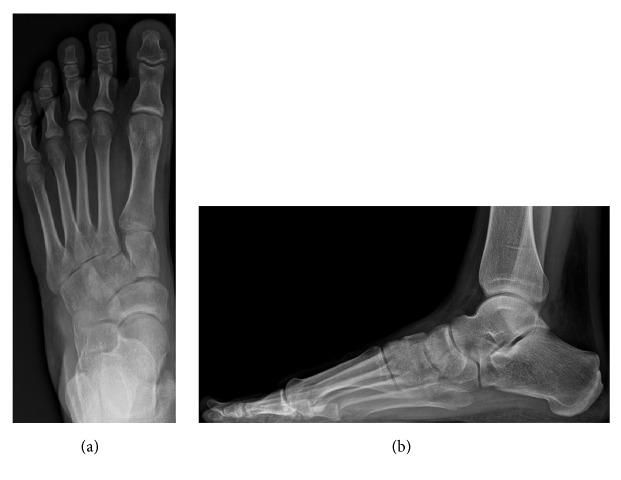
Preoperative anterior-posterior (a) and lateral (b) weight-bearing radiographs.

**Figure 6:**
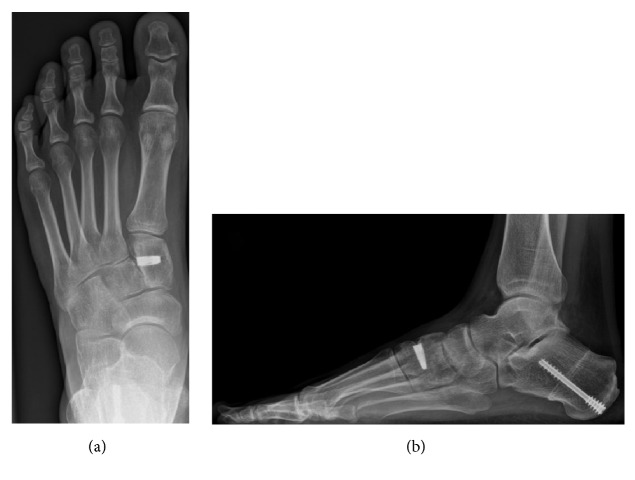
Postoperative (titanium wedge) anterior-posterior (a) and lateral (b) weight-bearing radiographs.

The next step was with patient placed in supine position. The Cotton osteotomy was performed with a 3 cm incision placed dorsally on the medial cuneiform and medial to the extensor halluces longus tendon. The tibialis anterior tendon is used intraoperatively as an anatomical reference point. The tibialis anterior tendon should be mobilized and retracted with its insertion protected throughout the procedure. At this point, the first metatarsocuneiform joint distally and the navicular-medial cuneiform joint proximally should be identified. Cotton osteotomy was performed at the midpoint of the medial cuneiform with an oscillating saw blade from dorsal to plantar, keeping the plantar cortex intact. The Hintermann distractor was then used to obtain a 4-6mm opening and checking the alignment between the forefoot and the hindfoot [10]. When the correction was achieved, the homologous bone graft was prepared, or the trabecular titanium wedge of the appropriate size, and positioned in the osteotomy site (Figures 1–3). In the HBG group, a temporary kirschner wire (KW) through the first cuneiform was used to provide stability. In the TTW group, no fixation was used (Figures 4–6).

### 2.2. Postoperative Care

The patient was placed in a short-leg nonweight-bearing cast for 4 weeks. Postoperative radiographs were assessed at 4 weeks after the surgery and, in the BWG, the temporary KW was removed. Partial weight-bearing without the cast was permitted when initial bone consolidation was present. Full unrestricted weight-bearing was allowed after 8 weeks.

### 2.3. Clinical and Radiographic Evaluation

**Figure 7:**
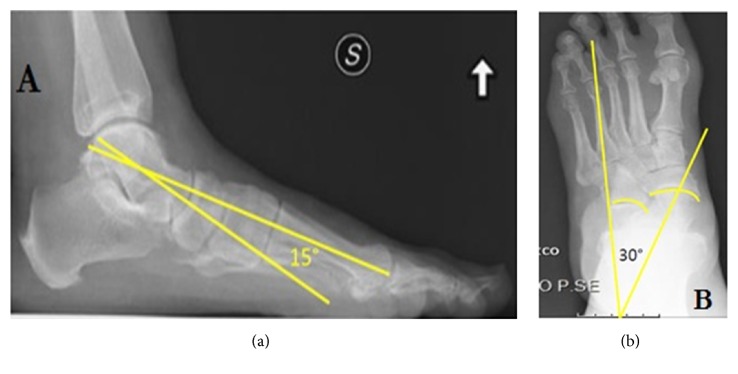
Preoperative radiographic evaluation. Meary's Angle (a) and Kite's Angle (b).

To eliminate surgeon bias, study investigations were conducted by an independent researcher (G.R.). The senior author (F.M.) was available to supervise each examination and investigation but did not examine the patients. The clinical evaluation was performed by means of different evaluation scales such as the American Orthopaedic Foot and Ankle Society (AOFAS) score [11, 12] and the Italian version of the Foot Function Index (FFI) [13]. Pain was quantified using a visual analogue scale (VAS) for pain from 0 to 10, with 0 representing no pain and 10, the worst pain imaginable [14]. Furthermore, patients subjectively evaluated the success of the operation with the following categories: “very satisfied,” “satisfied,” and “not satisfied”. Radiographic evaluation was performed in orthostatism with the Kite's Angle (talo-calcaneal; KA) and Meary's Angle (talus-first metatarsal; MA), before surgery and at the last follow-up (Figure 7) [15].

### 2.4. Statistical Analysis

Statistical analysis was performed using the software package SPSS, version 20 (IBM Corp., Armonk, NY). We compared differences in the clinical scores preoperatively and at the last follow-up with the paired Student's t-test for each group. Comparison of outcome scores between the groups was made using unpaired Student's t-test. Furthermore, we compared differences in the non-parametric variable (satisfaction rate) between the two groups using Pearson's chi-square test. A p value of less than 0.05 was considered statistically significant.

## 3. Results

None of the patients were lost to follow-up. Mean follow-up time for the TTW group was 21.7 ± 2.9 months (range, 18 to 25 months) and HBG group was 35.3 ± 8.5 months (range: 18 to 54 months). Mean age at surgery was similar between groups with patients in the TTW group 36.7 years-old (range: 18–64 years) and in the HBG group 38.5 years-old (range: 18–67 years) (p > 0.05). Concomitant surgical procedure was performed in both groups (Table 2).

Group outcomes are shown in Table 3. The difference between baseline and follow-up for both groups was statistically significant for all the clinical scores (p < 0.05). Based on the satisfaction rate, of the 18 patients in the TTW group, 16 (88.8%) were satisfied or very satisfied with the results compared with 17 of 18 (94.4%) patients in the HBG group (p >0.05).

KA and MA significantly improved in the two groups at final follow-up compared to those measured before surgery (p < 0.01 for both variables). KA and MA measured at the last follow-up were not significantly different between the two groups (Table 3).

In the TTW group, postoperative complications were recorded: one case of malpositioning of the titanium wedge, which required removal and bone graft to fill the gap, and one case of hallux valgus recurrence which required subsequent surgical correction.

In HBG group, postoperative complications were recorded: three cases of symptomatic bony prominence (one patient required surgical excision), one case of osteoarthritis of the first metatarsocuneiform joint (conservatively treated), and 1 case of injury of the terminal branch of the saphenous nerve which required neurectomy. No cases of osteolysis were recorded at the radiographic follow-up.

## 4. Discussion

Flatfoot is a multiplanar deformity that persists or develops after skeletal maturation and is characterized by complete or partial collapse of the medial longitudinal arch. Forefoot varus is commonly associated with flatfoot [16] and surgical correction should address this deformity [17]. Forefoot varus can be corrected through a plantar flexing opening wedge osteotomy of the medial cuneiform, also known as a Cotton osteotomy [6]. The first surgical technique performed with this procedure included the grafting of an autologous/homologous dorsal bone wedge to fill the osteotomy space. Different studies performed on this type of surgical technique evaluated the ability to correct the deformity by taking into account the restoration of the plantar arch, in particular the MA. In a study by Hirose et al. conducted on 16 patients, an improvement of 14° of the correction of the MA was observed. Furthermore, no cases of nonunion or residual pain were reported, reporting a 100% consolidation rate of cases [16].The high rate of consolidation depends on the blood supply of the medial cuneiform, provided by the medial tarsal artery, branch of the dorsal pedis artery [18]. Ling et al. tried to simplify the surgical technique by performing a wedge osteotomy with plantar subtraction ("reverse Cotton osteotomy") avoiding the surgical step of the bone graft [19]. Although the surgical technique included the medial incision, which may damage the medial tarsal artery, the results obtained in 10 patients reported a 100% osteotomy healing rate and an improvement of 10° of the MA. The importance of blood supply should be also considered when assessing the rates of nonunion for medial column arthrodesis: in previous papers, arthrodesis of the navicular cuneiform joint showed a variable nonunion rate between 11%-15% [20, 21] and the talonavicular arthrodesis 6.2% and the Lapidus arthrodesis 6.7% [22, 23]. Lutz et al. published a study with a large series of 81 patients treated with Cotton osteotomy in which was observed a 22° improvement for the MA [24]. However, most of the studies did not consider concurrent surgical steps for flatfoot correction and none reported clinical and functional assessment that took patient satisfaction into account. Our study showed that the Cotton osteotomy, when associated to different surgical steps, provided correction of the MA in both groups, comparable with previous studies.

Different complications related to the procedure are reported in the literature. In the study by Hirose et al., one patient had painful hardware that led to a revision surgery to remove the screws [16]. In the extensive series of Lutz et al., ten postoperative complications were reported in part attributable to Cotton osteotomy: 3 cases of painful hardware and consequent screw removal, 2 cases of dorsal exostosis, 1 case of painful sesamoid, 1 case of plantar fasciitis, 2 cases of lateral column overload, and 1 case of recurrence of flatfoot deformity [24]. Ling et al. reported in a small case series 1 case of hardware complication after a “reverse Cotton Osteotomy: the patient required hardware removal, tenosynovectomy at Henry's knot, and medial plantar neurolysis, resulting in good relief of symptoms. In this study, one patient in the TTW group required revision surgery, while two patients in the HBG group underwent revisional surgery [19].

Although the use of homologous/autologous bone grafts is still the most used in surgical practice, risks of disease transmission, donor site morbidity, and increase in nonunion rates should be taken into consideration. Furthermore, allografts in many countries are not available due to the lack of infrastructure, high costs, or religion, which may discourage the use of cadaveric tissues. Several devices have been recently developed to fill the opening osteotomy such as the trabecular titanium wedge-shaped materials capable of mimicking the structure of the cancellous bone and which are able to promote osseointegration [25–27]. The first study reported in the literature was performed by Gross et al. about 26 patients treated with the grafting of porous titanium wedges to lengthen the lateral column of the foot (Evans osteotomy) [25]. Radiographically, a significant correction of the deformity was reported with improvement of all angles of flatfoot in all patients. Only 1 patient reported nonunion of the osteotomy with fracture of the titanium wedge, which required subsequent removal and revision with iliac crest autograft; all the others (96%) showed complete osseointegration at the mean follow-up of 14.6 months. The use of a trabecular titanium wedge avoids side effect of residual pain at the donor site and ensures greater stability by eliminating the reabsorption phenomenon that sometimes occurs in bone grafts. However, a trabecular titanium wedge has not the structural characteristics of bone and may be subject to long-term mobilization or intolerance. Matthwes et al. in a recent paper reported clinical and radiological outcomes of patients treated with Cotton osteotomy, using a titanium wedge [28]. They found a 100% incorporation rate but one patient reported painful hardware. In our study, only one patient required revisional surgery in the TTW group and painful hardware was associated with malpositioning of the titanium wedge.

This is the first study to compare the clinical and radiological results of allograft to titanium trabecular wedge for forefoot varus correction. The results obtained show that the two techniques are similar, significantly improving all clinical and functional scores with high patient satisfaction in both groups. Despite no statistically significant differences were detected, complications were not similar. The HBG group reported a greater number of complications: 3 cases of dorsal exostosis, 1 case of osteoarthritis of the first metatarsocuneiform joint and 1 case of lesion of the terminal branch of the saphenous nerve that required subsequent neurectomy. The TTW group reported fewer complications: 1 case of implant malpositioning, which required removal and replacement with homologous bone, and 1 case of hallux valgus recurrence. The results of this study have further validated the findings of previous studies for titanium trabecular wedge, showing significant radiographic deformity correction with a favorable safety profile in patients with forefoot varus. The theoretical advantages of trabecular titanium wedges (i.e., no biological risks, no immune reactions, good availability, and no reabsorption phenomenon) make them a good alternative to traditional grafting materials.

## 5. Conclusions

The results in both clinical and radiographic terms showed that both techniques are comparable; most of patients were satisfied or very satisfied, with a low rate of complications. Although we believe that the average follow-up was long enough to detect implant-related soft tissue reactions, longer follow-up studies are needed to confirm trabecular titanium wedges osseointegration over time and its ability to maintain the correction.

## Figures and Tables

**Table 1:** Patient's demographic and radiographic data.

	TTW Group	HBG Group	P value
*Gender*			
Male	5 (38.5%)	8 (44.5%)	
Female	13 (61.5%)	10 (55.5%)	
*Age*	36.7 ± 17.2	38.5 ± 18.0	0.7

*AOFAS*	51.0 ± 20.7	61.0± 18.7	0.1
*FFI-Pain*	53.4 ± 20.2	47.3 ± 16.6	0.3
*FFI-Disability*	51.3 ± 23.3	45.3 ± 15.3	0.3
*VAS*	7.6 ± 1.9	7.2 ± 1.9	0.6
*KA (*°)	29.5 ±4.2	30.0 ± 4.0	0.7
*MA (*°)	9.5 ± 4.1	9.8 ± 4.0	0.8

**Table 2:** Concomitant Procedures performed at the index surgery.

Procedure	TTW	HBG
First metatarsal osteotomy	7	6
Gastrocnemius recession	9	13
Achilles Z lengthening	4	3

**Table 3:** Comparison of postoperative clinical scores and radiographic angles.

	TTW Group	HBG Group	p Value
AOFAS Score	90.2 ± 14.1	90.3 ± 13.9	0.8
FFI-Pain	20.5 ± 15.9	16.4 ± 15.8	0.4
FFI-Disability	21.6 ± 14.8	19.2 ± 17.5	0.6
VAS	3.3 ± 2.4	2.8 ± 2.4	0.4
KA (°)	21.5 ± 2.5	21.8 ± 2.4	0.6
MA (°)	1.4 ± 1.9	1.8 ± 1.7	0.5

## Data Availability

The datasets analysed during the current study are available from the corresponding author on reasonable request.
